# Coordinated silencing of the Sp1-mediated long noncoding RNA MEG3 by EZH2 and HDAC3 as a prognostic factor in pancreatic ductal adenocarcinoma

**DOI:** 10.20892/j.issn.2095-3941.2019.0427

**Published:** 2020-12-15

**Authors:** Ting Han, Meng Zhuo, Cuncun Yuan, Xiuying Xiao, Jiujie Cui, Guangrong Qin, Liwei Wang, Feng Jiao

**Affiliations:** 1 Department of Oncology, Renji Hospital, School of Medicine, Shanghai Jiaotong University, Shanghai 200127, China; 2 Department of Pathology, Fudan University Eye Ear Nose and Throat Hospital, Shanghai 201114, China; 3 Shanghai Center for Bioinformation Technology, Shanghai 201203, China; 4 State Key Laboratory of Oncogenes and Related Genes, Shanghai Cancer Institute, Renji Hospital, School of Medicine, Shanghai Jiaotong University, Shanghai 200127, China

**Keywords:** Pancreatic ductal adenocarcinoma, MEG3, Sp1, EZH2, HDAC3, miR-374a-5p

## Abstract

**Objective:**

Pancreatic ductal adenocarcinoma (PDAC) is a disease with high mortality. Many so-called “junk” noncoding RNAs need to be discovered in PDAC. The purpose of this study was therefore to investigate the function and regulatory mechanism of the long noncoding RNA MEG3 in PDAC.

**Methods:**

The Gene Expression Omnibus database (GEO database) was used to determine the differential expression of long noncoding RNAs in PDAC, and MEG3 was selected for subsequent verification. Tissue and cell samples were used to verify MEG3 expression, followed by functional detection *in vitro* and *in vivo*. Microarrays were used to characterize long noncoding RNA and mRNA expression profiles. Competing endogenous RNA analyses were used to detect differential MEG3 and relational miRNA expression in PDAC. Finally, promoter analyses were conducted to explain the downregulation of MEG3 PDAC.

**Results:**

We generated a catalogue of PDAC-associated long noncoding RNAs in the GEO database. The ectopic expression of MEG3 inhibited PDAC growth and metastasis *in vitro* and *in vivo*, which was statistically significant (*P* < 0.05). Microarray analysis showed that multiple microRNAs interacted with MEG3. We also showed that MEG3, as a competing endogenous RNA, directly sponged miR-374a-5p to regulate PTEN expression. The transcription factor, Sp1, recruited EZH2 and HDAC3 to the promoter and transcriptionally repressed MEG3 expression. Finally, clinical data showed that MEG3 and miR-374a-5p expressions were correlated with clinicopathological features. Statistically, Sp1, EZH2, HDAC3, and miR-374a-5p were negatively correlated with MEG3 (*P* < 0.05).

**Conclusions:**

Reduced MEG3 levels played a crucial role in the PDAC malignant phenotype, which provided insight into novel and effective molecular targets of MEG3 for pancreatic cancer treatment.

## Introduction

Pancreatic ductal adenocarcinoma (PDAC) is a global health challenge, with the fourth highest mortality and the twelfth highest morbidity^1^. The imbalance in morbidity and mortality arises from the difficulty in diagnosing the disease early and the rapid progression without obvious symptoms. A lack of novel predictable markers, a low resection rate, and chemoresistance have made it difficult to treat. Under such circumstances, a growing number of researchers are investigating the origins of PDAC carcinogenesis, what speeds its progression, and what mediates chemoresistance. An increasing number of studies are mainly focused on protein-coding genes, which follows the central dogma of “DNA-mRNA-protein.” However, tumor progression cannot be explicitly explained by only protein-coding genes, which only represent 1.5% of the whole genome^2^.

Recently, it has become clear that noncoding RNAs (ncRNAs) are of great importance in cancer initiation and progression. Similarly, long noncoding RNAs (lncRNAs), which are > 200 nucleotides in length, are one category of ncRNAs, which represent a new field for PDAC investigation^3^. HOTAIR^4^, MALAT-1^5–7^, H19^8^, HULC^9^, and PVT-1^10^ are highly expressed in pancreatic cancer, and each acts as an oncogene responsible for cancer development. However, few lncRNAs that act as tumor suppressors have been characterized. We screened microarrays that characterized the gene expression profiles between PDAC and normal pancreatic tissues from the GEO database and found a downregulated lncRNA, MEG3, to be a tumor suppressor.

We found decreased MEG3 in PDAC, and ectopic MEG3 expression inhibited cell growth and metastasis both *in vitro* and *in vivo*. We further showed that MEG3 could act as a competing endogenous RNA (ceRNA) to sponge miR-374a-5p. In addition, the low expression of MEG3 partly occurred through Sp1 transcriptional repression at the promoter region *via* interactions with EZH2 and HDAC3. Taken together, the results indicated that MEG3 played an important role in PDAC development, and could be a potential therapeutic target for PDAC patients.

## Materials and methods

### Ethics approval and consent to participate

Written informed consent was obtained from all patients, and the study was approved and supervised by the Ethics Committee of Renji Hospital, School of Medicine, Shanghai Jiaotong University (Approval No. RA-2019-121).

### Differentially expressed genes

We searched the Gene Expression Omnibus (GEO) DataSets in the NCBI portal (ncbi.nlm.nih.gov) using the key words of “Pancreatic Cancer and Normal” to compare the human PDAC tumor samples with normal samples. Two datasets, GSE15471 (78 PDAC tumors and matching normal pancreatic tissue samples) and GSE16515 (52 samples for both PDAC and normal samples) were found, which measured the gene expressions from large numbers of pancreatic tumors and normal tissues. From the literature survey, we also found the GSE32676 dataset, which measured the expression profiling of human PDAC (42 samples) and non-malignant pancreas samples (7 samples) using the same platform as GSE15471 and GSE16515. The large sample sizes for all 3 datasets allowed us to obtain a more robust result from differential expression analysis. Gene expression data were read using the Affy package^11^ in R. GCRMA^12^ was used to adjust for the background noise and normalize the gene expression data. Limma was used for the detection of differentially expressed genes. Differentially expressed probes were detected with a threshold *P* < 0.01 and an false discovery rate < 0.1, and then mapped to the gene level. Differentially expressed long noncoding genes were then filtered with the category defined in the HGNC database, which was downloaded from https://www.genenames.org/.

### Tissue samples and clinical data collection

A total of 70 paraffin-embedded tumor tissue samples and corresponding adjacent normal tissue samples were collected from the General Surgery Department, Renji Hospital, School of Medicine, Shanghai Jiaotong University. All tissues were independently confirmed to be PDAC by two pathologists. Eight fresh tumor samples and corresponding adjacent normal tissue samples were snap-frozen and stored in liquid nitrogen immediately after collection. None of the patients had any preoperative treatments, and the clinicopathological data were obtained from inpatient records.

### Cell line culture and reagents

Pancreatic cancer cell lines (BxPC-3, Capan-1, SW1990, AsPC-1, HPAF-II, and HS-766T) and the normal human pancreatic ductal epithelial cell line, HPDE, were all obtained from the Chinese Academy of Sciences Cell Bank (Shanghai, China). HS-766T cells were grown in 5% CO_2_ with saturated humidity at 37 °C, cultured in Dulbecco’s Modified Eagle’s Medium supplemented with 2 mmol/L glutamine and 10% fetal bovine serum (FBS) (both from Gibco, Gaithersburg, MD, USA) and subcultured by harvesting with trypsin-EDTA. HPDE, BxPC-3, CAPAN-1, AsPC-1, and HPAF-II cells were cultured in RPMI-1640 (Gibco) supplemented with 10% FBS. SW1990 cells were cultured in L-15 medium (Gibco) supplemented with 10% FBS. The 5-aza-2′-deoxycytidine was purchased from Sigma-Aldrich (St. Louis, MO, USA). Trichostatin A and 3-deazaneplanocin A (DZNeP) were purchased from Selleck Chemicals (Houston, TX, USA). Rabbit anti-Sp1, anti-EZH2, anti-Ago2, and anti-glyceraldehyde 3-phosphate dehydrogenase (GAPDH) were purchased from Cell Signaling Technology (Danvers, MA, USA). Rabbit anti-HDAC3 and anti-DNMT1 antibodies were purchased from Abcam (Cambridge, UK). Rabbit anti-β-actin was purchased from Sigma-Aldrich.

### Plasmid construction and cell transfection

The sequence of MEG3 was synthesized and subcloned into a pLenti-EF1a-EGFP-P2A-Puro-CMV-MCS vector (Obio Technology, Shanghai, China). Pancreatic cancer cells were infected with MEG3 or empty vector (NC). SiRNAs were obtained from GenePharma Biological Technology (Shanghai, China) and RebioBio (Guangzhou, China). The cells were ­ transfected with the siRNAs at 50% confluence using Lipofectamine Max (Invitrogen, Carlsbad, CA, USA) according to the ­manufacturer’s instructions. The sequences were as follows: EZH2: siRNA1, sense 5′-GGAUGGUACUUUCAUU GAATT-3′, antisense 5′-UUCAAUGAAAGUACCAUCCTT-3′; siRNA2, sense 5′-CGGCUUCCCAAUAACAGUATT-3′, ­antisense 5′-UACUGUUAUUGGGAAGCCGTT-3′; DNMT1: siRNA1, sense 5′-GCACCUCAUUUGCCGAAUATT-3′, antisense 5′-UAUUCGGCAAAUGAGGUGCTT-3′; siRNA2, sense 5′-GAGGCCUAUAAUGCAAAGATT-3′, antisense 5′-UCUUU GCAUUAUAGGCCUCTT-3′; miR-374a-5p mimics: sense 5′-UUUAUAAUACAACCUGAUAAGUG-3′, antisense 5′-CUU AUCAGGUUGUAUUAUAAUU-3′; miR-374a-5p inhibitor: 5′-CACUUAUCAGGUUGUAUUAUAA-3′; siRNA sequences targeting Sp1: siRNA1, 5′-CCAACAGATTATCACAAAT-3′; siRNA2, 5′-GGCTGGTGGTGATGGAATA-3′; siRNA3, 5′-GCC AATAGCTACTCAACTA-3′; siRNA sequences targeting HDAC3: siRNA1, 5′-CCATGACAATGACAAGGAA-3′; and siRNA2, 5′-GCATTGATGACCAGAGTTA-3′.

### RNA isolation and real-time quantitative polymerase chain reaction (qRT-PCR) analysis

Total RNA was isolated from cultured cells and tissue samples using a miniBEST Universal RNA Extraction Kit (Takara Bio, Mountain View, CA, USA) and an miRNeasy FFPE Kit (Qiagen, Hilden, Germany). Reverse transcription and RT-qPCR kits (Takara Bio) were used to evaluate genetic expression. The PCR primer pairs used for each gene were as follows: MEG3: sense 5′-GACATCATCCGTCCACCTCCTTG-3′, antisense 5′-GTCAGTGAGTGGCTGCTTTGTATG-3′; EZH2: sense 5′-C CGCAAGGGTAACAAAAT-3′, antisense 5′-GGTAGCAGAT GTCAAGGGA-3′; HDAC3, sense 5′-CCCGCATCGAGAATCA GAAC-3′, ­antisense 5′-TCAAAGATTGTCTGGCGGATCT-3′; DNMT1, sense 5′-GTGGGGGACTGTGTCTCTGT-3′, antisense 5′-TGAAAGCTGCATGTCCTCAC-3′; GAPDH, sense 5′-CCCC GCTACTCCTCCTCCTAAG-3′, antisense 5′-TCCACGA CCAGTTGTCCATTCC-3′; and miR-374a-5p, 5′-GGCGGTT ATAATACAACCTGATAAGTG-3′. The relative expression was calculated with the comparative threshold cycle (Ct) (2-ΔΔCt) method.

### Western blot analysis

Cells were lysed in RIPA lysis buffer, and the protein concentration was determined by a standard Bradford assay (Beyotime, Beijing, China). Equal amounts of protein (20 μg) from each cell line were subjected to Western blot analysis. Total proteins were fractionated using SDS-PAGE, transferred to polyvinylidene fluoride membranes, and incubated with the indicated primary and secondary antibodies. The data were adjusted against the loading controls of β-actin and GAPDH.

### Cell proliferation assay

The cell proliferation assay was performed using the SRB method. Stably transfected miR-374a-5p mimics and miR-inhibitor cells were seeded in 96-well microtiter tissue culture plates and cultured for 24 h, 48 h, and 72 h. At the end of the treatment, the cells were fixed with 10% w/v trichloroacetic acid (100 μL) for 1 h at 4 °C. The plates were then washed with deionized water and air dried. Samples were stained with 100 μL of SRB solution (in 0.4% w/v acetic acid) for 20 min at room temperature and then washed with acetic acid (1%) and air dried. Tris buffer (10 mM, 100 μL, pH 10) was added to each well for solubilization. Optical density values were measured at 540 nm with a reference wavelength of 630 nm using a microtiter plate reader (VersaMax; Molecular Devices, San Jose, CA, USA).

### Flow cytometry analysis of cell apoptosis and cell cycle

For cell apoptosis analysis, cells were harvested at 70%–80% confluence and incubated with reagent containing annexin V-fluorescein isothiocyanate and propidium iodide (BD Biosciences, San Jose, CA, USA) for 15 min in the dark at room temperature. Apoptotic cells were analyzed using a FACS Caliber flow cytometer (BD Biosciences).

### Colony formation and migration and invasion assays

For colony formation, 500 stably transfected cells were seeded in 6-well plates and maintained in complete medium for 2 weeks. Colonies were fixed with ice-cold methanol and stained with Crystal Violet. The cell invasive and migratory potentials were evaluated using Transwell assays (Millipore, Bedford, MA, USA). The cells were harvested from transfection in a 100 μL volume of serum-free medium and were placed in the upper chambers and incubated at 37 °C for less than 24 h. Then, they were stained with 0.1% Crystal Violet staining solution, and the cells on the upper surface of the membrane were removed. Counting was performed under a microscope in 5 randomly selected fields at a magnification of ×200 after drying. The details are provided in the Supplemental Information.

### Microarray analysis

SW1990-NC and MEG3 overexpression cells were used to detect the differential expression of microRNAs in triplicate experiments. The number of differentially expressed miRNAs for each comparison was calculated. Standard selection criteria to identify differentially expressed miRNAs included a *P* < 0.05 and a fold change > 2. The correlation of expression profiles between biological replicates and treatment conditions was determined by unsupervised hierarchical clustering analysis.

### Immunoprecipitation (IP)

Cells were collected and lysed using NP-40 (Beyotime). The total protein lysate (500 μg) was immunoprecipitated with an agarose-immobilized antibody (5 μg of anti-Sp1, -EZH2, -HDAC3, -HA, and -IgG antibodies) and incubated overnight at 4 °C. Protein A/G was added to the reaction, which was rotated for 4 h at 4 °C. The lysate was centrifuged at 3,000 rpm for 3 min, and the supernatant was discarded, followed by washing with NP-40, 10 times. Loading buffer (2×) was added to elute immune complexes from the beads, followed by immunoblot analysis.

### Chromatin immunoprecipitation (ChIP)

The ChIP kit was purchased from Cell Signaling Technology (Boston, MA, USA), and the experiments were performed according to the manufacturer’s instructions. Equal volumes of chromatin were immunoprecipitated with anti-Sp1, anti-EZH2, anti-HDAC3, and anti-trimethyl-histone H3 Lys27 (H3K27me3) or normal IgG as a negative control. Then, spin columns were used to purify DNA, followed by qPCR. The primers used for ChIP assays are listed as follows: promoter region (-2,000~-1,500): sense 5′-CAACTCATGCCCTTGTGGG-3′, antisense 5′-CGTGGGAACTGTGGTGGC-3′; promoter region (-1,500~-1,000): sense 5′-GCCTCTAGTGACCTGACGG-3′, antisense 5′-GGGAGATTCCAGGGCTGA-3′; promoter region (-1,000~-500): sense 5′-AGCTCAGGCATCTCCACA-3′, antisense 5′-CACCAGGGTTGTAATAAGAAT-3′; promoter region (-500~0): sense 5′-AAAAGCACCCGCGACCAC-3′, antisense 5′-GCAGCAAAGAAGGGAGGA-3′.

### Dual luciferase assay

A fragment containing MEG3 sequences from -2,000 bp relative to the transcription initiation site was subcloned into the pGL3-basic vector (Promega, Madison, WI, USA). The final full-length reporter plasmid, which contained multiple Sp1-binding sites, was designated pGL-MEG3. We also constructed segmental plasmids (pGL-MEG3-pro-2000, pGL-MEG3-pro-1500, pGL-MEG3-pro-1000, and pGL-MEG3-pro-500). Constructs were verified by sequencing of the inserts and flanking regions of the plasmids. We mutated the predicted binding sites: mut1: -2,000~0 bp, mut2: -1,500~0 bp, and mut3: ×1,000~0 bp, and mut4 was a combination of mut1, mut2, and mut3. Pancreatic cancer cells were transfected with the indicated MEG3 promoter reporter and expression plasmids. The MEG3 promoter activity was normalized *via* co-transfection of the empty vector. The luciferase activity in the cells was quantified using a dual luciferase assay system (Promega) 24 h after transfection.

### Real-time methylation-specific polymerase chain reaction (MSP-PCR)

We used the EpiTect MSP Kit (Qiagen) to perform the MSP-PCR. Primers were designed for the favored methylation site of the following MEG3 promoter sequences: MEG3-M, sense 5′-GTTAGTAATCGGGTTTGTCGGC-3′, antisense 5′-AATCATAACTCCGAACACCCGCG-3′; MEG3-U, sense 5′-GAGGATGGTTAGTTATTGGGGT-3′, and antisense 5′-CCACCATAACCAACACCCTATAATCACA-3′. Real-time PCR was performed at 95 °C for 30 s and 95 °C for 5 s, followed by 40 cycles of 57 °C for 20 s and 72 °C for 50 s. The real-time MSP products were evaluated using electrophoresis of standard 3% agarose gels, which were stained with ethidium bromide.

## Tumor formation and caudal vein injection model construction

The animal experiments were conducted according to the guidelines for animal experimentation and approved by the Experimental Animal Ethics Committee of Shanghai Jiaotong University (RA-2019-121). Five-week-old male athymic BALB/c mice were maintained under specific pathogen-free conditions and manipulated according to protocols approved by the Shanghai Medical Experimental Animal Care Commission. We harvested 1 × 10^7^ cells/mL for tumor formation and 5 × 10^6^ cells/mL for tail vein injection. Tumor volumes and weights were examined every week. Tumor volumes were measured as length × width^2^ × 0.5. After sacrifice, the specimens were kept on ice at 4 °C for further analysis.

### Statistical analysis

Statistical analysis was performed using SPSS statistical software for Windows, version 17.0 (SPSS, Chicago, IL, USA). For comparisons between two groups, we used a two-tailed Student’s *t*-test. Multiple group comparisons were calculated using one-way analysis of variance. The least-significant difference method was used for the post hoc test. The chi-square test was performed to identify associations between MEG3 expression levels and clinical characteristics. The survival curves were estimated using the Kaplan-Meier method. The log-rank test was used to estimate the significance of the differences between the survival curves. A Cox proportional hazards analysis was performed to calculate the hazard ratio (HR) and the 95% confidence interval (CI) to evaluate the association between MEG3 expression and overall survival (OS). A multivariate Cox regression was performed to adjust for other covariates. A *P*-value < 0.05 was considered statistically significant.

## Results

### Bioinformatics analysis of gene expression profiles showed that MEG3 was downregulated in PDAC

**Figure 1:**
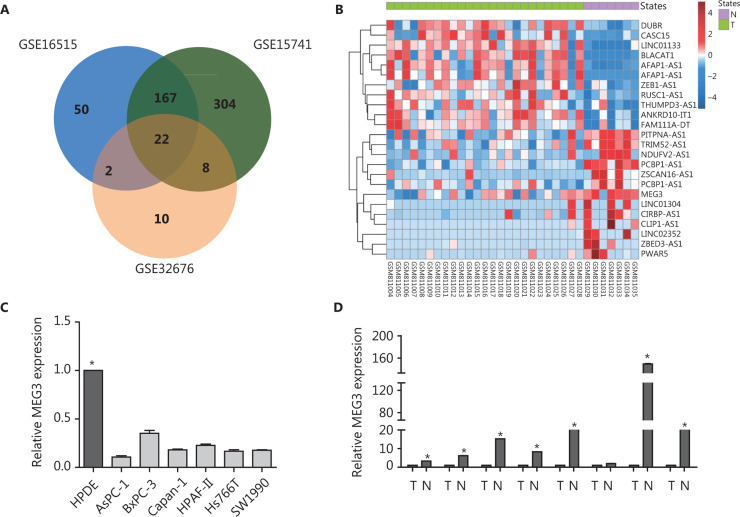
Differentially expressed long noncoding genes in pancreatic ductal adenocarcinoma (PDAC) compared with normal pancreatic tissue detected in multiple microarrays. (A) Venn diagram of the differentially expressed long noncoding genes in the three microarray datasets (GSE32676, GSE16515, and GSE15471). (B) Heat map of the differentially expressed long noncoding genes in the microarray dataset GSE32676. (C) Decreased MEG3 expression in PDAC cell lines, including AsPC-1, BxPC-3, Capan-1, HPAF-II, HS-766T, and SW1990 cells, compared to normal epithelial HPDE cells. (D) Decreased MEG3 expression in paired fresh PDAC tissue samples in comparison with normal tissues (*n* = 8). *P < 0.05.

To obtain an overview of the differential expression between PDAC and normal pancreatic tissue, we screened microarrays that measured gene expressions between PDAC and normal pancreatic tissues from the GEO database. Three microarrays from the GPL570 platform were selected, including GSE32676^13^, GSE16515^14^, and GSE15471^15^. Differentially expressed genes were detected using Limma^16^ as described in the Methods section. After filtering the differentially expressed long noncoding genes, we found 22 long noncoding genes that were differentially expressed between PDAC and normal pancreatic tissues, as shown in **Supplementary Table S1** and **Supplementary Figure S1**. The expression levels of the 22 long noncoding genes are shown in **Figure 1A and 1B**. PWAR5 and MEG3 were downregulated, and DUBR, CASC15, and BLACAT1 were upregulated in PDAC. MEG3 is a chromatin-interacting lncRNA that affects the expression of a wide range of target genes^17^. Furthermore, our data validated that MEG3 expression in PDAC cell lines was significantly decreased compared with that in normal pancreatic ductal epithelial (HPDE) cells, and the difference was statistically significant (**Figure 1C**). AsPC-1 and SW1990 showed lower expression of MEG3 than the other cell lines, so we used these two cell lines to conduct the following experiments. In addition, 8 paired fresh pancreatic cancer specimens showed decreased MEG3 expressions in PDAC tissues compared with adjacent normal tissues, and 7 of the 8 differences were statistically different (**Figure 1D**). These results demonstrated that MEG3 was expressed at lower levels in PDAC patients, when compared with adjacent normal tissues.

### MEG3 inhibited cell proliferation, growth, and metastasis *in vitro* and *in vivo*

**Figure 2:**
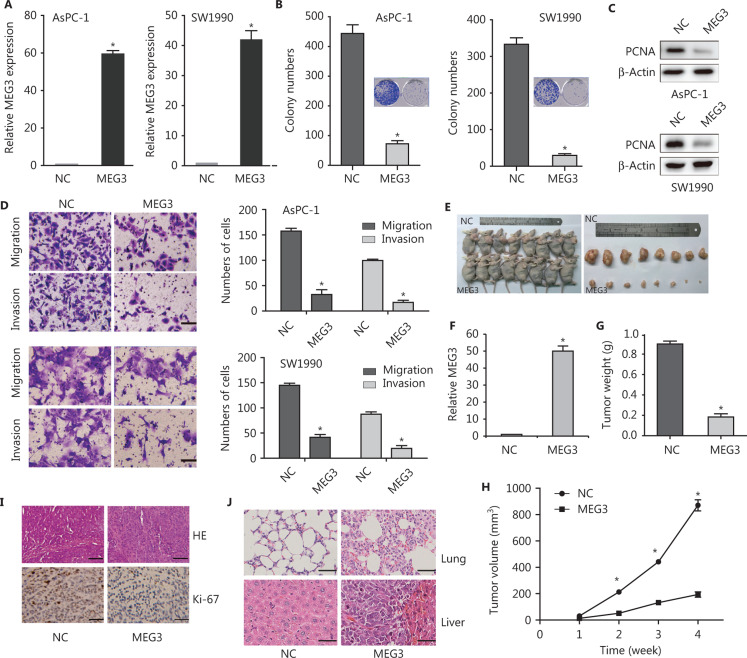
MEG3 inhibited cell growth and metastasis *in vitro* and *in vivo*. (A) Detection of MEG3 expression in stably transfected cells (AsPC-1 and SW1990 cells). (B) Colony numbers were lower in the MEG3 group than in the normal control (NC) group in colony formation assays. (C) Decreased PCNA expression in the MEG3 group was detected by Western blot. (D) Migration and invasion assays showed fewer cells in MEG3 group than in the NC group. Migration assay results: AsPC-1/NC: 153 ± 2.41 *vs*. MEG3: 21 ± 2.141; SW1990/NC: 169 ± 7.35 *vs*. MEG3: 23 ± 4.254; invasion assay results: AsPC-1/NC: 97 ± 5.52 *vs*. MEG3: 13 ± 1.241; SW1990/NC: 94.5 ± 4.95 *vs*. MEG3: 14 ± 1.414. (E) Effects of MEG3 overexpression on tumor growth *in vivo*. A representative image of xenografts is shown for the SW1990/MEG3 and normal control groups. (F) MEG3 expression was detected by qRT-PCR. (G) Tumor weights are represented as the means ± SD. (H) Tumor growth curves of the SW1990/NC and MEG3 groups. (I) Histopathology of xenograft tumors. The tumor sections were subjected to hematoxylin and eosin (HE) staining and immunohistochemical staining using antibodies against Ki-67. (J) HE staining showing the size and number of lung and liver metastases. *P < 0.05.

Because of the importance of low expression of MEG3 in PDAC progression, reactivation of MEG3 represented a promising therapeutic approach for PDAC. We constructed stable cell lines ectopically expressing MEG3 (**Figure 2A**). Colony formation assays showed that ectopic expression of MEG3 inhibited cell growth (**Figure 2B**). The expression of PCNA protein, which reflected cell proliferation, decreased significantly in the MEG3 group (**Figure 2C**). The number of migrated (AsPC-1/NC: 153 ± 2.41 *vs.* MEG3: 21 ± 2.141; SW1990/NC: 169 ± 7.35 *vs.* MEG3: 23 ± 4.254) and invaded (AsPC-1/NC: 97 ± 5.52 *vs.* MEG3: 13 ± 1.241; SW1990/NC: 94.5 ± 4.95 *vs.* MEG3: 14 ± 1.414) cells was significantly lower when MEG3 was upregulated (**Figure 2D**).

We also determined whether MEG3 could inhibit PDAC growth *in vivo*. Ectopic expression of MEG3 in SW1990 cells inhibited tumor growth *in vivo* (**Figure 2E**). Tumor size and weight were remarkably reduced compared with those in the control group (**Figure 2F and 2G**). Hemotoxylin and eosin (HE) staining of subcutaneously transplanted tumors in nude mice showed dense cells, poor differentiation, decreased gland and tumor nest distributions, increased mitosis, obvious heteromorphosis, and partial necrosis (**Figure 2H**). Ki-67 expression was indeed significantly decreased in the MEG3 groups (**Figure 2H**), indicating that the tumor suppression by MEG3 was at least partially mediated through proliferation inhibition.

Metastasis xenografts were established by injections through the caudal vein. HE staining showed tumors with concentrated staining and disrupted polarities in the corresponding lung tissues. The size and number of lung metastases in the MEG3 group were significantly smaller than those in the control group (**Figure 2I**). Taken together, these results suggested that MEG3 inhibited tumor growth and metastasis *in vivo*.

### MiR-374a-5p reversed the inhibitory effects of MEG3 in PDAC

**Figure 3:**
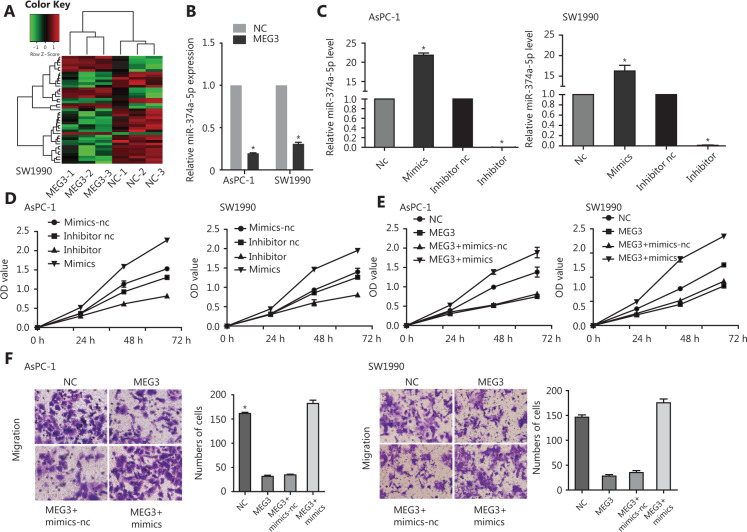
MiR-374a-5p served as an oncogene and reversed the effects of MEG3 on pancreatic ductal adenocarcinoma cells. (A) A microarray was used to analyze the constructed normal control (NC) and MEG3 overexpression cell lines. (B) The miR-374a-5p expression in the NC and MEG3 groups. (C) AsPC-1 and SW1990 cells that were transfected with miR-374a-5p mimics or inhibitor were subjected to qRT-PCR for miR-374a-5p expression. (D) Overexpression of miR-374a-5p promoted cell proliferation, while downregulation of miR-374a-5p inhibited cell proliferation. (E) Overexpression of miR-374a-5p enhanced the inhibitory effect of MEG3 on cell proliferation. (F) Overexpression of miR-374a-5p enhanced the inhibitory effect of MEG3 on cell migration. *P < 0.05.

Microarray analysis was performed using the constructed NC and MEG3 overexpression PDAC cell lines (**Supplementary Table S2**). We found that there were 41 microRNAs with different levels of expression: 13 were upregulated and 28 were downregulated (**Figure 3A**). The downregulation of miR-374a-5p occurred the most. To verify its expression in PDAC, ectopic expression of MEG3 was examined, and it was found to be associated with decreased miR-374a-5p expression (**Figure 3B**). To confirm the biological function of miR-374a-5p in PDAC, we detected cell proliferation after miR-374a-5p overexpression or inhibition (**Figure 3C**). Cell proliferation was increased by miR-374a-5p overexpression, while miR-374a-5p knockdown showed the opposite effect (**Figure 3D**). These results indicated that miR-374a-5p served as an oncogene in PDAC. To dissect the importance of miR-374a-5p binding in promoting PDAC progression, we ectopically expressed miR-374a-5p in stable MEG3-overexpressing cells. We found that overexpression of miR-374a-5p enhanced the inhibitory effect of MEG3 on cell proliferation (**Figure 3E**) and migration (**Figure 3F**). These findings indicated that MEG3 promoted PDAC cell proliferation and migration, in part by competitively binding with miR-374a-5p.

### MEG3 directly sponged miR-374a-5p to regulate PTEN expression in PDAC

**Figure 4:**
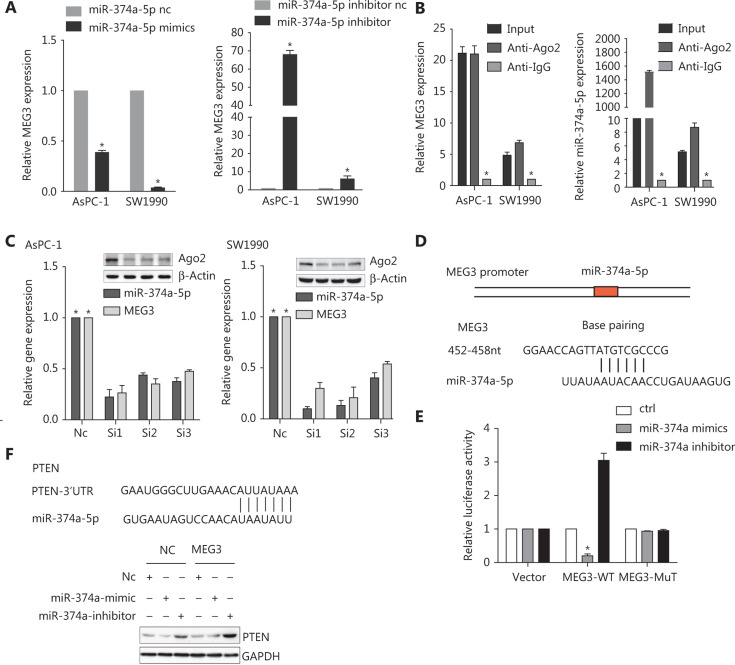
MEG3 directly sponged miR-374a-5p to regulate PTEN expression in pancreatic ductal adenocarcinoma. (A) MEG3 expression was detected in cells transfected with miR-374a-5p mimics or inhibitor. (B) A RIP assay was performed, and the co-precipitated RNA was subjected to qRT-PCR. RNA levels were presented as fold enrichments in Ago2 relative to IgG immunoprecipitates. (C) Relative MEG3 and miR-374a-5p expressions were detected after Ago2 precipitation. (D) A sequence alignment of miR-374a-5p with the binding sites of MEG3 is shown. (E) The relative luciferase activity of 293T cells was tested after co-transfection with MEG3 wild-type/mutant plasmids and miR-374a-5p mimics or inhibitor. (F) PTEN expression was detected in cells after co-transfection with NC/MEG3 plasmids and miR-374a mimic/inhibitor. *P < 0.05.

To investigate the potential interaction between MEG3 and miR-374a-5p, we transfected PDAC cell lines with miR-374a-5p mimics or miR-inhibitors; MEG3 expression was decreased or increased, respectively (**Figure 4A**). RNA Binding Protein Immunoprecipitation experiments were performed and RISC complexes were found to precipitate after administration of anti-Ago2 antibodies. As a result, MEG3 and miR-374a-5p were successfully enriched in cell lines by administration of anti-Ago2 antibodies, when compared with IgG antibodies (**Figure 4B**). Additionally, silencing Ago2 decreased MEG3 and miR-374a-5p expressions in PDAC cell lines (**Figure 4C**). Taken together, these results indicated that miR-374a-5p and MEG3 existed together in complexes.

Next, we used bioinformatics to predict the binding sites of MEG3 and miR-374a-5p by using lnCeDB (http://gyanxetbeta.com/lncedb/) (**Figure 4D**). Compared with the negative control group, the group that was transfected with miR-374a-5p mimics had a significantly reduced intensity of the fluorescence signal representing the full-length MEG3 reporter gene. After miR-374a-5p inhibitor transfection, the intensity of the fluorescence signal was significantly upregulated, while mutation of the binding site partially inhibited this effect (**Figure 4E**). The results indicated that MEG3 directly bound to miR-374a-5p. Subsequently, we used TargetScan and miRanda to predict the downstream target genes of miR-374a-5p, and we validated the PTEN gene in combination with a literature survey. The expression of PTEN was downregulated after transfection with miR-374a-5p mimics in the MEG3-NC group; however, PTEN was increased in the MEG3 ectopic expression group (**Figure 4F**). Transfection of the miR-374a-5p inhibitor into the ectopic MEG3 expression group largely increased PTEN expression (**Figure 4F**). Taken together, these results suggested that MEG3 can be used as a ceRNA to regulate the occurrence and progression of PDAC.

### Suppression of Sp1, EZH2, or HDAC3 level led to reduced MEG3 expression

**Figure 5:**
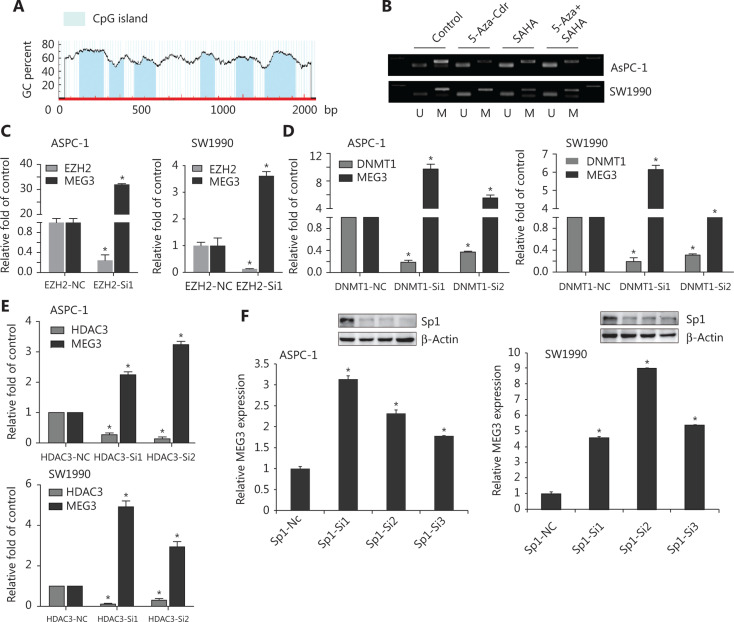
Suppression of Sp1, EZH2, or HDAC3 levels led to reduced MEG3 expression. (A) The promoter region of MEG3 was rich in multiple CpG islands. (B) Expression of methylated and unmethylated MEG3 in pancreatic ductal adenocarcinoma cells treated with 5-AZA-CdR, SAHA or their combination. (C) MEG3 expression in EHZ2-nc and EZH2-siRNA cells. (D) MEG3 expression in DNMT1-nc and DNMT1-siRNA cells. (E) MEG3 expression in HDAC3-nc and HDAC3-siRNA cells. (F) MEG3 expression in Sp1-nc and Sp1-siRNA cells. *P < 0.05.

We next investigated the epigenetic regulation of MEG3 through histone acetylation and methylation. Using the Li Lab website (http://www.urogene.org/cgi-bin/methprimer/methprimer.cgi), it was predicted that the promoter region of MEG3 was rich in multiple CpG islands (**Figure 5A**). A previous study showed that hypermethylation in the differential methylation region was closely related to MEG3 silencing in tumorigenesis^18^. Our group previously examined whether DNA methylation inhibitors (5-Aza-CdR), histone methyltransferase inhibitors (DZNeP), and HDAC inhibitors (SAHA) could lead to increased MEG3 expression^19^. The PCR products of the nonmethylated MEG3 promoter increased after drug treatment (**Figure 5B**). Overall, these observations implied that both HDACs and PRC2 were involved in MEG3 expression.

EZH2, as well as HDAC3, promoted PDAC invasion and metastasis^20,21^. We determined whether EZH2 and HDAC3 acted together to affect MEG3 expression in PDAC. The roles of EZH2 and HDAC3 in the transcriptional regulation of MEG3 was first examined by depleting their expressions. In agreement with our earlier study, silencing of EZH2, DNMT1, and HDAC3 upregulated MEG3 expression **(Figure 5C, 5D and 5E)**.

Histone modification requires transcription factors for downstream target gene transcriptional regulation^22,23^. There are multiple transcription factor binding sites in the MEG3 promoter region, including a binding site for Sp1. In our previous study, Sp1 was highly expressed in PDAC and promoted cell invasion and progression^24^. Notably, we found that knockdown of Sp1 upregulated MEG3 expression (**Figure 5F**).

### Sp1, EZH2, and HDAC3 were tethered to MEG3 promoter regions as a corepressor complex to inhibit MEG3 transcription

**Figure 6:**
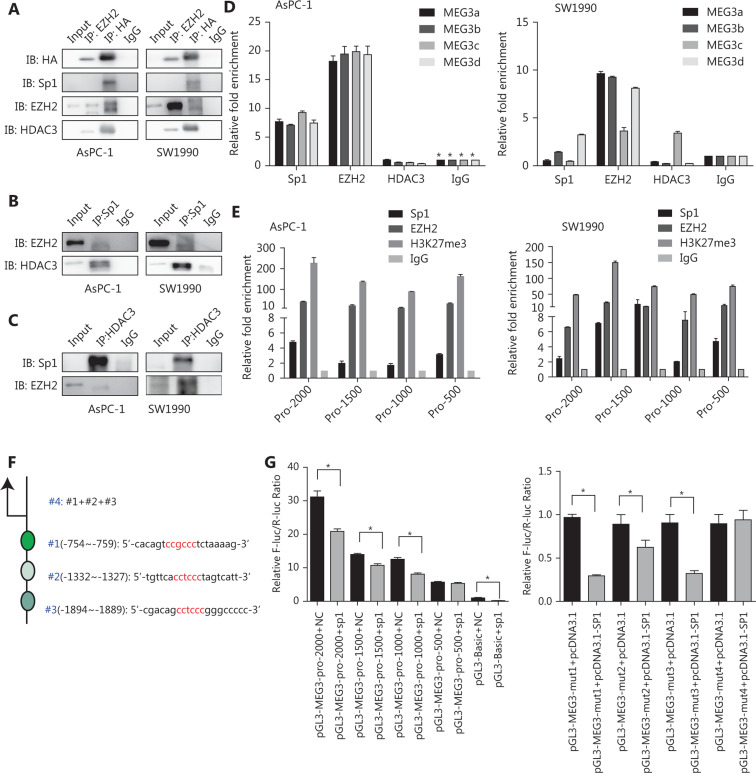
Sp1, EZH2, and HDAC3 are tethered to MEG3 promoter regions as a corepressor complex to inhibit MEG3 expression. (A) The 293T cells were transfected with the HA-HDAC3 plasmid. Whole cell lysates were immunoprecipitated using antibodies against EZH2, HA, and control IgG, followed by Western blot analysis with antibodies against HA, Sp1, EZH2, and HDAC3. (B) Cell lysates were immunoprecipitated with antibodies against Sp1 and control IgG, followed by Western blot analysis with antibodies against EZH2 and HDAC3. (C) Cell lysates were immunoprecipitated with antibodies against HDAC3 and control IgG, followed by Western blot analysis with antibodies against EZH2 and Sp1. (D) ChIP assay showing Sp1 and EZH2 enrichment on MEG3 promoters. (E) Segmental chromatin immunoprecipitation assay showing Sp1 and EZH2 enrichment on MEG3 promoters in each 500 bp region. (F) Mutated locations of the MEG3 promoter (depletion mutation). (G) Luciferase activities showing that expression of wild-type MEG3 promoter was repressed by Sp1 overexpression in the -2,000~-500 bp region but not in the -500–0 bp region.

We then investigated whether EZH2 and HDAC3 formed a corepressor complex with Sp1 to repress MEG3 expression using co-immunoprecipitation assays. First, 293T cells were co-transfected with vectors expressing HA-tagged full-length HDAC3, and the presence of Sp1, HDAC3, and EZH2 was detected in the immunoprecipitates obtained with antibodies against HA and EZH2. When immunoprecipitated with anti-Sp1, EZH2 and HDAC3 were detected, while immunoprecipitation with anti-HDAC3 resulted in the coprecipitation of Sp1 and EZH2 (**Figure 6A, 6B, and 6C**).

Next, we examined the MEG3 promoter regions for transcription factor binding sites and identified highly conserved Sp1 binding sites in a region ~2 kb upstream of the transcription start site. We investigated whether EZH2 and HDAC3 could be recruited to the MEG3 promoter by Sp1. We used primers located within the MEG3 proximal promoter regions of Sp1 binding sites and showed that antibodies against both EZH2 and Sp1 efficiently immunoprecipitated the MEG3 ­promoter regions, but HDAC3 could not bind directly (**Figure 6D**). Bioinformatics software prediction analysis showed that the MEG3 promoter region contained multiple Sp1 binding sites, and every 500 bp had a Sp1 binding site. Therefore, aiming at the MEG3 promoter region of -2000–0 bp, the MEG3 promoter was divided into 500 bp segments, and primers were designed to predict binding sites. Segmental CHIP experiments showed that the binding sites of Sp1 and EZH2 in the MEG3 promoter region were consistent with those predicted (**Figure 6E**). To test whether the Sp1 binding was functional, we generated luciferase reporter constructs carrying the wild-type and mutated promoter segments of MEG3. The results indicated that luciferase activities of the wild-type -2,000~ -500 bp MEG3 promoter region was significantly repressed by Sp1 overexpression, while the -500–0 bp promoter region activity was unaffected. The mut1 (-2,000~0 bp), -mut2 (1,500~0 bp), and mut3 (-1000~0 bp) promoter regions were used to predict the effects of mutations in the promoter, and the three segments were combined and named mut4 (**Figure 6F**). Compared with wild-type promoters, the luciferase activity of the mutated promoters was not significantly changed by Sp1 overexpression (**Figure 6G**). These results were consistent with those of the ChIP experiments showing the strongest binding of Sp1 in MEG3 promoter. Overall, these results showed that the MEG3 loci contained Sp1-binding regions that were under negative control by HDAC3 and EZH2, and that histone hypoacetylation and trimethylation contributed to Sp1-induced MEG3 repression.

### MEG3 was associated with prognosis and inversely correlated with EZH2, HDAC3, Sp1, and miR-374a-5p

Finally, we investigated the correlation between MEG3 and clinicopathological features in PDAC patients. Expression of both MEG3 and miR-374a-5p was correlated with clinical stage and infiltration depth, and these correlations were statistically significant. In addition, miR-374a-5p was statistically correlated with tumor location, lymph node metastasis, and perineural invasion (**Supplementary Table S3**).

Next, we used the Kaplan-Meier method and a Cox regression model to analyze the relationship between MEG3/miR-374a-5p expression and prognoses in PDAC patients. MEG3 and miR-374a-5p were closely related to the prognoses of patients with PDAC. Patients with higher MEG3 and lower miR-374a-5p expression had better prognoses than patients with lower MEG3 and higher miR-374a-5p expression (**Supplementary Figure S2A and S2B**). Multivariate analysis suggested that MEG3 and miR-374a-5p could be regarded as independent factors for PDAC patients (**Table 1**).

**Table 1:** Univariate and multivariate analysis of prognostic factors in pancreatic ductal adenocarcinoma

Clinicopathological parameters	Univariate analysis			Multivariate analysis		
	HR	95% CI	*P*	HR	95% CI	*P*
MEG3 expression						
High	1					
Low	2.805	1.108–7.105	0.011	2.932	1.149–7.484	0.018*
miR-374a-5p expression						
High	3.292					
Low	1	1.206–8.990	0.02	6.393	1.115–36.649	0.037*
Age (years)						
≤ 60	1					
> 60	1.129	0.483–2.639	0.779			
Gender						
Male	1					
Female	0.819	0.370–2.193	0.819			
Tumor location						
Head, neck	1					
Body, tail	1.227	0.698–2.159	0.477			
Tumor size (cm)						
≤ 3	0.75					
> 3	1	0.336–1.675	0.481			
Invasion depth						
T1 + T2	1					
T3 + T4	1.429	0.566–3.604	0.447			
Lymph nodes metastasis						
N0 (negative)	1					
N1 (positive)	0.933	0.408–2.133	0.87			
Clinical stage						
Early stages (≤ II)	1			1		
Advanced stages (> II)	2.55	1.112–5.851	0.018	2.703	1.164–6.276	0.021*
Nervous system invasion						
Negative	1					
Positive	1.048	0.447–2.453	0.915			

HR, hazard ratio; 95% CI. 95% confidence interval, *P < 0.05.

After analyzing their expression and correlation, Sp1, EZH2, HDAC3, and miR-374a-5p were found to be negatively correlated with MEG3, and these correlations were statistically significant (**Supplementary Figure S2C, S2D, S2E and S2F**).

## Discussion

Pervasive lncRNAs are reported in various tumors, acting as either oncogenes^25,26^ or tumor suppressor genes^27^. Although numerous well-known lncRNAs have been identified and have been investigated for their functions in different kinds of tumors, the expression of lncRNAs is strikingly cell type-specific in normal tissues^28–30^ and different cancer types^31^. We screened microarrays that measured the gene expression profiles between PDAC and normal pancreatic tissues from the GEO database, and MEG3 was found to be downregulated. A MEG3 gene transcribes a 1.6 kb lncRNA, which acts as an antitumor component in different cancer cells^32,33^, such as breast, liver, glioma, colorectal, cervical, gastric, lung, and ovarian and osteosarcoma cancer cells. The biological function of MEG3 to repress tumors was through regulating the major tumor suppressor genes, *p53* and Rb, inhibiting angiogenesis-related factor, or controlling miRNAs^32^. In our PDAC samples, MEG3 was also decreased. Ectopic MEG3 expression inhibited cell growth and metastasis *in vivo* and *in vitro*, partially through G2/M arrest and apoptosis. This result is consistent with that of a previous report^34,35^. P53 plays an important role in maintaining G2/M cycle arrest and inducing apoptosis^36^. It has been reported that MEG3 plays a role in suppressing cancer by upregulating p53^37^. In PDAC, we previously found that p53 was upregulated in cell lines that were treated with 5-AZA-CdR, which also upregulated MEG3 expression^19^.

LncRNAs can regulate genetic expression at the transcriptional and post-transcriptional levels. LncRNAs do not function independently. They are mainly regulated by the binding of other DNAs, RNAs, or proteins. The ceRNA hypothesis presents an alternative function for lncRNAs^38^. A novel regulatory mechanism has been identified in the crosstalk between lncRNAs and mRNAs. Song et al.^39^ confirmed that the lncRNA HOTAIR promoted gastric cancer progression by negatively regulating miR-152. In PDAC, it is possible that ectopic expression of MEG3 inhibits tumor invasion and metastasis by regulating miRNA expression. Our microarray analysis showed that 41 differentially expressed microRNAs, and miR-374a-5p were decreased the most. Previous studies have reported that miR-374a-5p serves as a key inhibitory miRNA during the invasion and migration of tumors by regulating the related genes of the wnt/β-catenin signaling pathway^40^. Our current research showed an inverse correlation between MEG3 and miR-374a-5p. Additionally, we found an endogenous interaction between MEG3 and miR-374a-5p, which functioned together to regulate PTEN expression.

EZH2, as a transcriptional inhibitor, participates in cancer development. The frequent EZH2 overexpression found in human cancers is associated with more aggressive cancer phenotypes with poor prognosis. EZH2 can promote the development and evolution of prostate cancer by regulating HK2-mediated tumor anaerobic glycolysis^41^. In addition, EZH2 also plays an important role in promoting lung cancer evolution by interacting with multiple lncRNAs, such as lncRNA 00511^42^, NEAT^43^, and TUG1^44^. Mondal et al.^17^ identified 276 lncRNAs enriched on chromosomes affected by EZH2 inhibition by ChIP-seq, including MEG3. Histone methylation and histone deacetylation are related to the transcriptional activity of the gene. A number of studies have shown that EZH2 can interact with HDACs to inhibit the expression of the downstream target genes^45,46^. Our previous study found that EZH2 and HDAC3 were negatively correlated with the prognoses of pancreatic cancer patients^20,21^. These reports agree with our previous and current findings that MEG3 expression is upregulated through silencing of EZH2 and HDAC3. In addition, EZH2 requires a cooperating transcription factor to transcriptionally regulate downstream target genes^22,23,47^. By analyzing the MEG promoter region, we found that there were multiple transcriptional factors that bound to the MEG3 promoter region, including Sp1. Sp1 was the first cloned and purified nuclear transcription factor, and it plays an important role in tumor growth, metastasis and angiogenesis by regulating downstream target genes, and is highly expressed in PDAC and associated with poor prognosis^24^. With the development of epigenetic technology, numerous studies have confirmed that transcription factors can recruit methylation-related enzymes and acetyltransferase, allowing for regulation of downstream target genes^48^. The latest research shows that Sp1 not only regulates the DNA methyltransferase DNMT1^49^, but also recruits epigenetically related co-enzymes, such as HDAC1^50^, HDAC2, and participates in gene methylation modification, acting as transcriptional activators or inhibitors^51–53^. Here, we revealed that Sp1 cooperated with EZH2 and HDAC3 to transcriptionally repress MEG3 expression. Finally, we validated gene expression and clinical features in human pancreatic cancer patients. MEG3 and miR-374a-5p expressions were correlated with clinical stage and infiltration depth, which could be prognostic factors for PDAC patients. In addition, Sp1, EZH2, HDAC3, and miR-374a-5p were negatively correlated with MEG3.

## Conclusions

**Figure 7:**
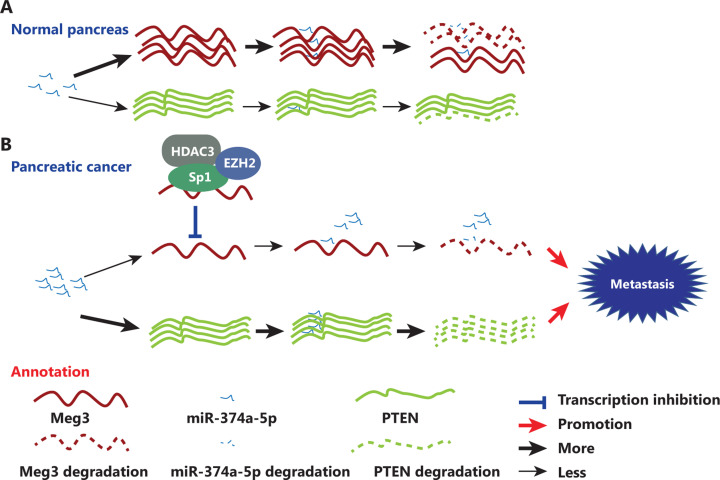
Schematic illustration of the coordinated silencing of the Sp1-mediated long noncoding RNA MEG3 by EZH2 and HDAC3 in pancreatic ductal adenocarcinoma. (A) Normal MEG3 regulation in normal pancreas. (B) Sp1 recruits EZH2 and HDAC3 to the MEG3 promoter and transcriptionally represses its expression. MEG3 directly sponges miR-374a-5p to regulate PTEN expression in PDAC.

MEG3 has been identified as a potential suppressor of several types of tumors, but the delivery of lncRNAs remains problematic, limiting its applications. Considering all these results, we propose a model in which Sp1 mediates EZH2/HDAC3 recruitment to repress MEG3, which can sponge miR-374a-5p to regulate PTEN expression in PDAC (**Figure 7A and 7B**). Our significant identification of the Sp1-EZH2/HDAC3-MEG3-miR-374a-5p axis provides insight into how MEG3 is inactivated and contributes to the aggressive transformation of tumors, thus revealing mechanistic links between MEG3 and its upstream and downstream signaling in PDAC.
