# Supplementary material for: Coordinated silencing of the Sp1-mediated long noncoding RNA MEG3 by EZH2 and HDAC3 as a prognostic factor in pancreatic ductal adenocarcinoma
Source: Cancer Biol Med. 2020 Dec 15;17(4):953–69. doi: 10.20892/j.issn.2095-3941.2019.0427 (PMC7721101; doi:10.20892/j.issn.2095-3941.2019.0427)
Supplement: Supplementary file 1 [file cbm-17-953-s001.pdf]

Supplementary materials

Table S1 Differentially expressed long noncoding genes in pancreatic ductal adenocarcinoma compared with normal pancreatic tissues

Gene	Smallest adjusted *P*-value	Log (FC)	Up/Down
TRIM52-AS1	9.399968e-08	−1.0354805	Down
PITPNA-AS1	2.808495e-07	−1.080543	Down
LINC02352	3.530234e-07	−0.8114819	Down
PWAR5	4.686704e-06	−1.152608	Down
ZSCAN16-AS1	8.39662e-05	−0.4721906	Down
NDUFV2-AS1	0.0001000207	−0.6264711	Down
ZBED3-AS1	0.0001446375	−0.4846355	Down
CIRBP-AS1	0.0002131723	−0.536308	Down
PCBP1-AS1	0.0002247987	−0.8636266	Down
LINC01304	0.0002259213	−0.3518953	Down
MEG3	0.002217913	−0.2342561	Down
CLIP1-AS1	0.007525541	−0.2447061	Down
DUBR	4.732483e-11	1.4446612	Up
CASC15	2.402067e-10	1.079979	Up
ZEB1-AS1	5.05486e-09	1.07719	Up
ANKRD10-IT1	1.087309e-08	1.318941	Up
BLACAT1	2.582637e-07	2.405326	Up
AFAP1-AS1	8.248845e-07	1.357795	Up
THUMPD3-AS1	1.614973e-06	1.2725470	Up
FAM111A-DT	1.699994e-06	1.160243	Up
LINC01133	2.87819e-05	3.247532	Up
RUSC1-AS1	0.008604316	0.6451321	Up

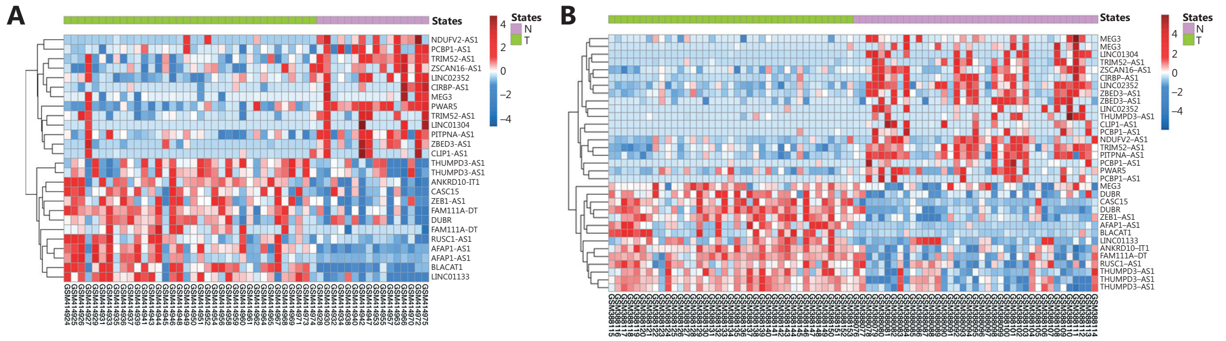

Figure S1 Differentially expressed long noncoding genes in the GSE16515 and GSE15471 datasets. (A) Heat map of the differentially expressed long noncoding genes in the microarray dataset, GSE16515. (B) Heat map of the differentially expressed long noncoding genes in the microarray dataset, GSE15471.

Table S2 A microarray analysis was performed using the constructed normal control and MEG3 overexpression pancreatic ductal adenocarcinoma cell lines

Name	*P*-value	Log2 ratio	Up/Down
hsa-miR-575	0.021512	1.633021923	Up
hsa-miR-758-5p	0.031165	1.501874765	Up
hsa-miR-660-5p	0.000264	1.089782218	Up
hsa-miR-34c-3p	0.017906	1.464105941	Up
hsa-miR-7856-5p	0.026255	1.041659152	Up
hsa-miR-422a	0.034919	1.249385605	Up
hsa-miR-378a-3p	0.034017	1.453833702	Up
hsa-miR-4423-3p	0.046857	1.084779057	Up
hsa-miR-6509-5p	0.048009	1.121458814	Up
hsa-miR-4428	0.04401	1.527293274	Up
hsa-miR-3120-3p	0.031732	1.160711181	Up
hsa-miR-4476	0.030771	1.000794592	Up
hsa-miR-130b-3p	0.010843	1.0648644	Up
hsa-miR-5008-3p	0.032333	−1.57723	Down
hsa-miR-526b-5p	0.044232	−1.01641	Down
hsa-miR-625-3p	0.017976	−1.01295	Down
hsa-miR-3158-3p	0.017772	−1.52853	Down
hsa-miR-4704-3p	0.029385	−1.50174	Down
hsa-miR-6719-3p	0.021194	−2.05628	Down
hsa-miR-6806-3p	0.043104	−2.34648	Down
hsa-miR-3975	0.017284	−1.11221	Down
hsa-let-7c-5p	0.022587	−1.17341	Down
hsa-miR-4699-3p	0.027858	−2.43855	Down
hsa-miR-3613-3p	0.046182	−1.93161	Down
hsa-miR-3118	0.005871	−1.58821	Down
hsa-miR-4775	0.048858	−2.16352	Down
hsa-miR-1296-5p	0.018411	−1.0925	Down
hsa-miR-374a-5p	0.040316	−5.61353	Down
hsa-miR-3611	0.019591	−5.34521	Down
hsa-miR-93-3p	0.008226	−1.2408	Down
hsa-miR-4785	0.017286	−1.20483	Down
hsa-miR-423-3p	0.027608	−1.10063	Down
hsa-miR-6502-5p	0.032829	−1.16439	Down
hsa-miR-103a-2-5p	0.014402	−1.01893	Down
hsa-miR-4455	0.015509	−1.21161	Down
hsa-miR-4694-5p	0.019766	−2.79507	Down
hsa-miR-3672	0.01518	−1.28064	Down
hsa-miR-4701-3p	0.004724	−1.0486	Down
hsa-miR-5587-5p	0.017929	−1.18744	Down
hsa-miR-576-5p	0.039621	−2.85526	Down
hsa-miR-4700-3p	0.046985	−1.31126	Down

Continued

Table S3 Associations between MEG3/miR-374a-5p expression and clinicopathological characteristics in pancreatic cancer

Clinicopathological parameters	No. of patients	MEG3 expression		*P*-value	miR-374a-5p expression		*P*-value
		High (%)	Low (%)		High (%)	Low (%)	
Cases	70	35 (50.0)	35 (50.0)		42 (60.0)	28 (40.0)	
Age (years)							
≤ 60	29	15 (53.6)	14 (46.4)	0.812	20 (69.0)	9 (31.0)	0.198
> 60	41	19 (47.9)	22 (52.1)		22 (53.7)	19 (46.3)	
Sex							
Male	44	20 (45.8)	24 (54.2)	0.476	28 (63.6)	16 (36.4)	0.419
Female	26	15 (57.1)	11 (42.9)		14 (53.8)	12 (46.2)	
Tumor location							
Head, neck	51	28 (54.5)	23 (45.5)	0.305	35 (68.6)	16 (31.4)	0.016*
Body, tail	19	7 (38.1)	12 (61.9)		7 (36.8)	12 (63.2)	
Tumor size (cm)							
≤ 2	26	12 (46.2)	14 (53.8)	0.809	16 (61.5)	10 (38.5)	0.84
> 2	44	23 (52.0)	21 (48.0)		26 (59.1)	18 (40.9)	
Invasion depth							
T1 + T2	13	10 (81.5)	3 (18.5)	0.000*	2 (15.4)	11 (84.6)	0.000*
T3 + T4	57	20 (32.7)	37 (67.3)		40 (70.2)	17 (29.8)	
LN metastasis							
N0 (negative)	19	13 (64.3)	6 (35.7)	0.095	7 (35.3)	12 (64.7)	0.023*
N1 (positive)	51	23 (41.7)	28 (58.3)		34 (66.7)	17 (33.3)	
Clinical stage							
Early stages (≤ II)	51	35 (66.7)	16 (33.3)	0.000*	17 (33.3)	34 (66.7)	0.023*
Advanced stages (> II)	19	1 (5.3)	18 (94.7)		11 (64.7)	6 (35.3)	
Perineural invasion							
Negative	22	7 (33.3)	15 (66.7)	0.083	9 (40.9)	13 (59.1)	0.027*
Positive	48	28 (57.7)	20 (42.3)		33 (68.8)	15 (31.2)	

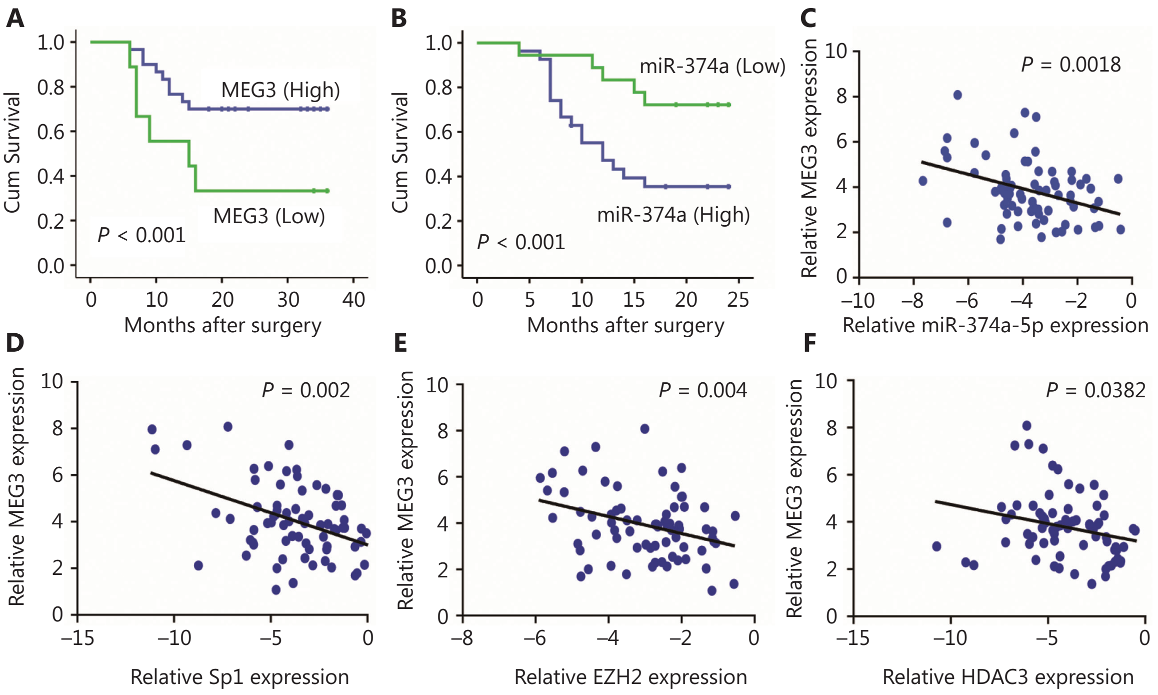

Figure S2 MEG3 was associated with prognosis and inversely correlated with EZH2, HDAC3, Sp1, and miR-374a-5p. (A) Kaplan-Meier analysis of MEG3 in pancreatic ductal adenocarcinoma patients. Higher expression of MEG3 favored a better prognosis. (B) Kaplan-Meier analysis of miR-374a-5p in PDAC patients. Lower expression of miR-374a-5p favored a better prognosis. (C) A negative relationship between MEG3 and Sp1 was revealed by Pearson's correlation analysis. (D) A negative relationship between MEG3 and EZH2 was revealed by Pearson's correlation analysis. (E) A negative relationship between MEG3 and HDAC3 was revealed by Pearson's correlation analysis. (F) A negative relationship between MEG3 and miR-374a-5p was revealed by Pearson's correlation analysis.

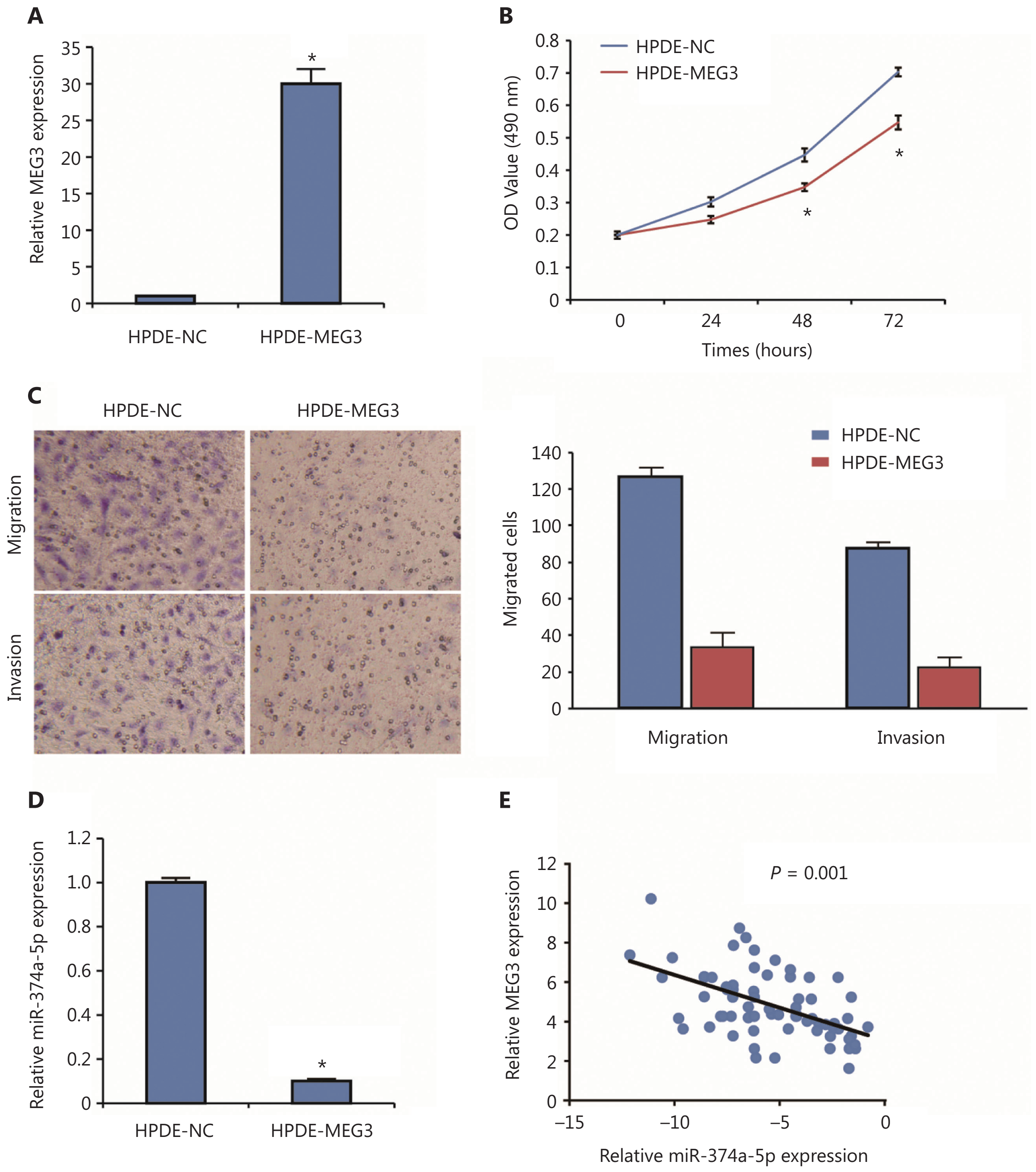

Figure S3 MEG3 and miR-374a-5p expression in HPDE cells and normal pancreatic tissues. (A) MEG3 expression was detected by qRT-PCR in HPDE cells after MEG3 overexpression. (B, C) MEG3 overexpression promoted cell proliferation (B) migration, and invasion (C) in HDPE. (D) MEG3 overexpression in HPDE resulted in downregulation of miRNA-374a-5p. (E) MEG3 and miR-374a-5p were negatively correlated in normal pancreas tissues (n = 70).

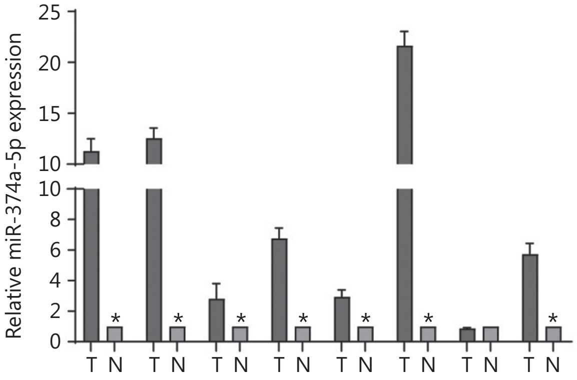

Figure S4 Relative miR-374a-5p expression in paired fresh normal and pancreatic ductal adenocarcinoma tissue samples (n = 8).
